# Privacy-Preserving Average-Tracking Control for Multi-Agent Systems with Constant Reference Signals

**DOI:** 10.3390/e28010120

**Published:** 2026-01-19

**Authors:** Wei Jiang, Cheng-Lin Liu

**Affiliations:** 1 School of Mechanical, Electrical and Information Engineering, Wuxi Vocational Institute of Arts & Technology, Wuxi 214206, China; 2 School of Internet of Things Engineering, Jiangnan University, Wuxi 214122, China; liucl@jiangnan.edu.cn

**Keywords:** constant reference signals, privacy-preserving, integral-type average-tracking algorithm, mismatched reference signals

## Abstract

This paper addresses the average-tracking control problem for multi-agent systems subject to constant reference signals. By introducing auxiliary signals generated from the states and delayed states of agents, a novel privacy-preserving integral-type average-tracking algorithm is proposed. Leveraging the frequency-domain analysis approach, delay-dependent sufficient and necessary conditions for ensuring asymptotic average-tracking convergence are derived. Furthermore, the proposed algorithm is extended to tackle the average-tracking control problem with mismatched reference signals, and a corresponding delay-dependent sufficient condition is established to guarantee privacy-preserving average-tracking convergence. Numerical simulations are conducted to verify the effectiveness of the developed algorithms.

## 1. Introduction

In recent decades, the average-tracking control problem of multi-agent systems, where each agent has a different reference signal, has attracted much attention due to its wide applications in distributed estimation and tracking, distributed resource allocation, sensor fusion, map merging, etc. [1].

For multi-agent systems where each agent is assigned an individual reference signal, the average-tracking control problem requires that each agent asymptotically converge to the average value of all reference signals through a distributed control protocol. To address the average-tracking control problem with constant reference signals, various proportional–integral (PI) average-tracking control algorithms have been proposed and analyzed for first-order multi-agent systems [2,3,4,5], with the necessary convergence condition being that the information-exchanging topology is balanced. Considering agents subject to heterogeneous external disturbances, Liu et al. integrated disturbance observers into the PI average-tracking control algorithm and derived the necessary and sufficient conditions for the system with identical time delays and a balanced topology [6]. For multi-agent systems tasked with tracking heterogeneous time-varying reference signals, numerous sophisticated average-tracking control algorithms have been developed and theoretically verified to be effective [7,8,9,10].

Notably, increasing attention has been devoted to multi-agent systems with mismatched reference signals, where valid agents are equipped with individual reference signals, while the remaining auxiliary connecting agents have no reference signals. Evidently, the average-tracking control problem for such systems poses greater challenges in analysis and synthesis. For first-order multi-agent systems with mismatched reference signals, Shan and Liu proposed a novel PI average-tracking control algorithm and derived a delay-dependent convergence condition for the system with identical time delays [11]. In addition, Chung and Kia adopted novel PI average-tracking control algorithms for first-order agents subject to mismatched time-varying reference signals, though non-negligible tracking errors persisted [12]. For the average-tracking control problem where mismatched constant reference signals are set as the initial states of the corresponding agents, Shao and Tian developed a constructive consensus algorithm under the premise that each agent must acquire the total number of agents and the number of reference signals [13]. Considering heterogeneous linear multi-agent systems, Liu et al. [14] designed a hierarchical average-tracking control algorithm to address the average-tracking problem with mismatched reference signals, which consists of an average-consensus algorithm and a decentralized tracking controller. Furthermore, Liu et al. analyzed the convergence conditions under directed and balanced topologies by leveraging the graph theory and the matrix theory [14].

In the coordination control of multi-agent systems, privacy preservation is consistently required due to inter-agent communication. Specifically, the privacy-preserving average-consensus problem, where each agent asymptotically converges to the average value of all agents’ initial states, has garnered significant research attention. To safeguard the privacy of initial states, obfuscation signals are incorporated into the agents’ dynamics and transmitted information [15,16]. Additionally, Wang [17] proposed a privacy-preserving average-consensus algorithm based on state decomposition, wherein each agent’s state is partitioned into two sub-states: one sub-state is updated via coordination control using neighbors’ corresponding sub-states, while the other sub-state evolves through internal computations. This state decomposition strategy thereby ensures the privacy of initial states [17]. Furthermore, other privacy-preserving techniques, such as homomorphic encryption [18] and differential privacy [19], have also been adopted in the consensus control of multi-agent systems.

In this paper, a privacy-preserving average-tracking control algorithm is proposed for first-order multi-agent systems with constant reference signals. The key design idea lies in constructing auxiliary signals that are distinct from the agents’ states and reference signals, with only these auxiliary variables transmitted for inter-agent coordination control. Firstly, the proposed algorithm is analyzed under the scenario of matched constant reference signals, and a delay-dependent sufficient and necessary convergence condition is derived via the frequency-domain analysis approach. Subsequently, for the case of mismatched constant reference signals, the proposed algorithm is modified, and a corresponding delay-dependent convergence condition is obtained based on frequency-domain analysis.

**Notation.** *R*, $R^{p}$, and $R^{p\times q}$ represent the set of real numbers, *p*-dimensional real vectors, and p×q real matrices, respectively. $1_{n}={\left[1,1,\cdots ,1\right]}^{T}$ denotes the *n*-dimensional column vector with all elements of 1, and $I_{n}$ represents a n×n identity matrix. Let R(s) be the set of all polynomials (rational functions, respectively) in *s* with real coefficients, and let $R{\left(s\right)}^{p\times q}$ be p×q complex matrices with elements in R(s). *C* denotes the set of complex numbers. For a matrix $P\in C^{n\times n}$, det(P) is the determinant of *P*, and ρ(P) is the matrix spectral radius. For $q_{1}\in C,q_{2}\in C$, $Co(q_{1},q_{2})$ is the convex hull of two complex numbers. For any $a_{1},\cdots ,a_{n}\in C$, $\mathrm{diag}\{a_{1},\cdots ,a_{n}\}$ is a diagonal matrix with diagonal elements $a_{1},\cdots ,a_{n}$.

## 2. Problem Description

### 2.1. Preliminaries

Considering the following first-order multi-agent system

(1) $${\dot{x}}_{i}\left(t\right)=u_{i}\left(t\right),i\in N,$$

where N={1,⋯,n}, and $x_{i}\left(t\right)\in R$ and $u_{i}\left(t\right)\in R$ denote the state and control input, respectively.

**Average-tracking control problem.** Each agent has a constant reference signal $r_{i}\in R$, and the control goal of agents (1) is

(2) $$\lim_{t\to \infty }x_{i}\left(t\right)=\frac{1}{n}\sum_{k\in N}r_{k},i\in N.$$

The information-exchanging topology of system (1) is depicted as an undirected graph G=(N,E,A), and G consists of a node set N, a edge set E⊆N×N, and a matrix $A=\left[a_{i,j}\right]\in R^{n\times n}$ with adjacent weight $a_{ij}\geq 0$. $e_{i,j}=\left(i,j\right)\in E$ is an undirected edge between *i* and *j* and implies that each node can receive information from the other one. $N_{i}=\left\{j\in N:e_{i,j}\in E\right\}$ denotes the neighbor set of node *i*. Suppose that $a_{ij}=a_{ji}>0\Leftrightarrow e_{i,j}\in E$ and $a_{i,i}=0$ for all i∈N. Then, we define $L=D-A=\left[l_{i,j}\right]\in R^{n\times n}$ as the Laplacian matrix and $D=\mathrm{diag}\{\sum_{j=1}^{n}a_{i,j},i\in N\}$ as the degree matrix. A path between the nodes $i_{s}$ and node $i_{e}$ is a sequence of edges $\left(i_{s},i_{2}\right),\cdots ,\left(i_{s-1},i_{e}\right)$. The undirected graph is connected if each node has a path to every other node.

In this paper, we focus on the undirected graph as follows.

**Assumption** **1.** *The information-exchanging topology G of system (1) is undirected and connected.*

### 2.2. Critical Lemmas

**Lemma** **1** ([20,21])**.** *L has a simple eigenvalue of *0*, and $L1_{n}=0$ if the corresponding topology G is undirected and connected.*

According to Assumption 1 and Lemma 1, we list the eigenvalues of *L* as $\lambda_{1}=0,\lambda_{2},\cdots ,\lambda_{n}$, and $\lambda_{i}>0,i=2,\cdots ,n$.

**Lemma** **2** ([22])**.** *Consider the proper transfer function matrix $H\left(s\right)\in R{\left(s\right)}^{M\times M}$, where M is a positive integrator. Φ(s)=det(I+H(s)) has its zeros lying in the open left-half complex plane if and only if the number of encircling (−1,j0) counterclockwise by the eigenloci of H(jω), ω∈(−∞,+∞), is equivalent to the number of poles lying in the right-half plane of H(s).*

**Lemma** **3** ([23])**.** *Define $W\in C^{n\times n}$, $W=W^{★}\geq 0$ and $Q=\mathrm{diag}\{q_{i},q_{i}\in C,i\in N\}$. Then,*

$$\lambda \left(WQ\right)\in \rho \left(W\right)Co(0\cup \left\{q_{i}\right\}),$$

**Lemma** **4.** *Let $\phi_{1}\left(j\omega \right)=\frac{1-\mu e^{-j\omega \tau }}{-\omega^{2}+j\kappa \omega }$ with κ>0 and 1>μ>0. Assume that $\phi_{1}\left(j\omega \right)=\frac{1-\mu e^{-j\omega \tau }}{-\omega^{2}}$ with ω>0 crosses the negative real axis for the first time at $\omega_{c1}$. Then, $\omega_{c1}>\frac{\pi }{\tau }$ and satisfies*

(3) $$\mu \omega_{c1}\sin\left(\omega_{c1}\tau \right)+\kappa (1-\mu \cos\left(\omega_{c1}\tau \right))=0.$$

*In addition, $\phi_{1}\left(j\omega \right)$ with $\omega \in (0,\omega_{c1})$ is in the third quadrant.*

**Proof.**  Based on the expression of $\phi_{1}\left(j\omega \right)$, we obtain

(4) $$\arg\left(\phi_{1}\left(j\omega \right)\right)=-\pi +\arctan\left(\frac{\mu \sin(\omega \tau )}{1-\mu \cos(\omega \tau )}\right)+\arctan\left(\frac{\kappa }{\omega }\right),$$

where arg(·) denotes the phase. According to the definition of $\omega_{c1}$, we obtain

$$\arg\left(\phi_{1}\left(j\omega \right)\right)<-\pi ,\omega \in \left(0,\omega_{c1}\right),\;\mathrm{and}\;\arg\left(\phi_{1}\left(j\omega_{c1}\right)\right)=-\pi .$$

Obviously, $\phi_{1}\left(j\omega \right)$ lies in the third quadrant for $\omega \in (0,\omega_{c1})$. □

**Lemma** **5.** *Let $\phi_{2}\left(j\omega \right)=\frac{1-\mu e^{-j\omega \tau }}{-\omega^{2}}$ with κ>0 and 1>μ>0.*

*1.*  *$\phi_{2}\left(j\omega \right)$ is in the third quadrant with $\omega \in (0,\omega_{c2})$ and $\omega_{c2}=\frac{\pi }{\tau }$;*
*2.*  *$|\phi_{1}\left(j\omega \right)|<|\phi_{2}\left(j\omega \right)|\leq \frac{1+\mu }{\omega_{c2}^{2}}$ with $\omega \in [\omega_{c2},+\infty )$.*

**Proof.**  From the expression of $\phi_{2}\left(j\omega \right)$, we obtain

(5) $$\arg\left(\phi_{2}\left(j\omega \right)\right)=-\pi +\arctan\left(\frac{\mu \sin(\omega \tau )}{1-\mu \cos(\omega \tau )}\right),$$

and it is obvious that

(6) $$\arg\left(\phi_{1}\left(j\omega \right)\right)>\arg\left(\phi_{2}\left(j\omega \right)\right).$$

Additionally, $\phi_{2}\left(j\omega \right)$ passes through the negative real axis at $\omega_{c}=\frac{\pi }{\tau }$ for the first time. Hence, it is concluded that $\phi_{2}\left(j\omega \right)$ with $\omega \in (0,\omega_{c})$ is in the third quadrant.

Then, by computation, we obtain

(7) $$|\phi_{2}\left(j\omega \right)|=\frac{|1-\mu e^{-j\omega \tau }|}{\omega^{2}}.$$

Obviously, we have

(8) $$|\phi_{1}\left(j\omega \right)|<|\phi_{2}\left(j\omega \right)|=\frac{\sqrt{1^{2}+\mu^{2}-2\mu \cos\left(\omega \tau \right)}}{\omega^{2}}\leq \frac{1+\mu }{\omega^{2}}.$$

Hence, $|\phi_{1}\left(j\omega \right)|<|\phi_{2}\left(j\omega \right)|\leq \frac{1+\mu }{\omega_{c}^{2}}=\frac{\left(1+\mu \right)\tau^{2}}{\pi^{2}}$ also holds with $\omega \in [\omega_{c},+\infty )$. □

## 3. Privacy-Preserving Average-Tracking Algorithm

Referring to the PI average-tracking algorithm in [12], we propose the following privacy-preserving average-tracking algorithm:

(9) $$\begin{array}{ccc} u_{i}\left(t\right) & = & \kappa \left(r_{i}-x_{i}\left(t\right)\right)+\kappa_{I}\sum_{j\in N_{i}}a_{ij}\left(z_{j}\left(t\right)-z_{i}\left(t\right)\right),\\ {\dot{z}}_{i}\left(t\right) & = & \sum_{j\in N_{i}}a_{ij}\left(\phi_{j}\left(t\right)-\phi_{i}\left(t\right)\right),i\in N, \end{array}$$

where κ>0, $\kappa_{I}>0$, $a_{ij}>0$ is the coupling weight, and $z_{i}\left(t\right)\in R$ and $\phi_{i}\left(t\right)\in R$ are the auxiliary variables.

**Remark** **1.** *In algorithm (9), the signals $z_{i}\left(t\right)$ and $\phi_{i}\left(t\right)$ are transmitted through a communication network so as to reach the average-tracking collective behavior. In this paper, $\phi_{i}\left(t\right)=f\left(x_{i}\left(t\right),x_{i}\left(t-\tau \right)\right)\in R$ is designed as a function for the state $x_{i}\left(t\right)$ and its delayed state $x_{i}\left(t-\tau \right)$, where τ>0 is the time delay. Obviously, the privacy is preserved because the states $x_{i}\left(t\right)$ cannot be directly obtained from the signals $z_{i}\left(t\right)$ and $\phi_{i}\left(t\right)$. Compared with existing privacy-preserving mechanisms, including those based on external signal introduction [15,16,19], the Paillier cryptosystem [18] and state decomposition [17], the proposed privacy-preserving algorithm exhibits distinct simplicity. This advantage stems from the fact that no complex external signals are incorporated into the agents’ dynamics, and the system dimension remains unchanged.*

With algorithm (9), the closed-loop form of agents (1) is

(10) $$\begin{array}{ccc} {\dot{x}}_{i}\left(t\right) & = & \kappa \left(r_{i}-x_{i}\left(t\right)\right)+\kappa_{I}\sum_{j\in N_{i}}a_{ij}\left(z_{j}\left(t\right)-z_{i}\left(t\right)\right),\\ {\dot{z}}_{i}\left(t\right) & = & \sum_{j\in N_{i}}a_{ij}\left(\phi_{j}\left(t\right)-\phi_{i}\left(t\right)\right),i\in N. \end{array}$$

In this paper, we adopt

(11) $$\phi_{i}\left(t\right)=x_{i}\left(t\right)-\mu x_{i}\left(t-\tau \right),$$

where μ∈(0,1), τ>0 is time delay, and the system (10) becomes

(12) $$\begin{array}{ccc} {\dot{x}}_{i}\left(t\right) & = & \kappa \left(r_{i}-x_{i}\left(t\right)\right)+\kappa_{I}\sum_{j\in N_{i}}a_{ij}\left(z_{j}\left(t\right)-z_{i}\left(t\right)\right),\\ {\dot{z}}_{i}\left(t\right) & = & \sum_{j\in N_{i}}a_{ij}\left(\left(x_{j}\left(t\right)-\mu x_{j}\left(t-\tau \right)\right)-\left(x_{i}\left(t\right)-\mu x_{i}\left(t-\tau \right)\right)\right),i\in N. \end{array}$$

By adopting the Laplace transform, we obtain the characteristic equation of system (12) for $x\left(t\right)={\left[x_{1}\left(t\right),\cdots ,x_{n}\left(t\right)\right]}^{T}$ is

(13) $$\det(s^{2}I+\kappa s+\kappa_{I}g\left(s\right)L^{2})=0,$$

where $g\left(s\right)=1-\mu e^{-s\tau }$.

**Theorem** **1.** *Investigate the multi-agent system (12), and the information-exchanging topology satisfies Assumption 1. If and only if*

(14) $$\kappa_{I}\lambda_{\max}^{2}\frac{1+\mu }{\omega_{c1}\sqrt{\omega_{c1}^{2}+\kappa^{2}}}<1$$

*holds, where $\omega_{c1}$ satisfies (3) and $\lambda_{\max}=\max_{i\in N}\lambda_{i}$, the agents (12) reach the average value of the reference signal asymptotically.*

**Proof.**  Let $f\left(s\right)=\det(s^{2}I+\kappa s+\kappa_{I}g\left(s\right)L^{2})$. It follows from Lemma 1 and Assumption 1 that f(0)=0 and f(s) has one simple zero at s=0. □

Considering s≠0, Equation (13) can be rewritten as

(15) $$\det(I+\kappa_{I}\hat{g}\left(s\right)L^{2})=0,$$

which equals

(16) $$\prod_{i=1}^{n}\left(1+\kappa_{I}\lambda_{i}^{2}\hat{g}\left(s\right)\right)=0,$$

where $\hat{g}\left(s\right)=\frac{1-\mu e^{-s\tau }}{s(s+\kappa )}$, and $\lambda_{1}=0,\lambda_{i}>0,i=2,\cdots ,n$. Obviously, (16) is equivalent to

(17) $$1+\kappa_{I}\lambda_{i}^{2}\hat{g}\left(s\right)=0,i=2,\cdots ,n.$$

Equation (17) has its roots lying in the open left-half complex plane if and only if $\kappa_{I}\lambda_{i}^{2}\hat{g}\left(j\omega \right)$ with ω>0 does not enclose (−1,j0). From Lemma 4, $\kappa_{I}\lambda_{i}^{2}\hat{g}\left(j\omega \right)$ passes through the negative real axis at $\omega_{c1}$ in (3) for the first time. Hence, $\kappa_{I}\lambda_{i}^{2}\hat{g}\left(j\omega \right)$ with ω>0 does not enclose (−1,j0) if and only if (14) holds.

Consequently, Equation (13) has its roots lying in the open left-half complex plane except for one root at s=0, i.e., $\lim_{t\to \infty }x_{i}\left(t\right)=x_{i}^{*},i\in N,\lim_{t\to \infty }z_{i}\left(t\right)=z_{i}^{*},i\in N$. Hence, it follows from dynamical Equation (5) that $L{\left[x_{1}^{*},\cdots ,x_{n}^{*}\right]}^{T}=0$. Using Lemma 1, it can be concluded that $x^{*}={\left[x_{1}^{*},\cdots ,x_{n}^{*}\right]}^{T}=c1_{n}$, i.e., the system (12) asymptotically achieves stationary consensus.

Then, one obtains

$$0=\kappa \left(r_{i}-x_{i}\left(t\right)\right)+\kappa_{I}\sum_{j\in N_{i}}a_{ij}\left(z_{j}\left(t\right)-z_{i}\left(t\right)\right).$$

Assumption 1 guarantees the symmetry of the adjacent weights, and it results in

$$\sum_{i=1}^{n}\kappa \left(r_{i}-c\right)=0,$$

which yields

$$c=\frac{1}{\sum_{i=1}^{n}}\sum_{i=1}^{n}r_{i}=\frac{1}{n}\sum_{k=1}^{n}r_{k}.$$

Therefore, the agents (12) asymptotically reach the average value of reference signals. Theorem 1 is proved.

Moreover, $\omega_{c1}>\frac{\pi }{\tau }$ in Lemma 4 yields the following sufficient condition.

**Corollary** **1.** *Consider the multi-agent system (12) under an information-exchanging topology that satisfies Assumption 1. The agents (12) asymptotically converge to the average value of reference signals, if*

(18) $$\tau <\sqrt{\frac{2\pi^{2}}{\sqrt{\kappa^{4}+4{\left(\kappa_{I}\lambda_{\max}^{2}\left(1+\mu \right)\right)}^{2}}-\kappa^{2}}}$$

*holds.*

**Figure 1:**
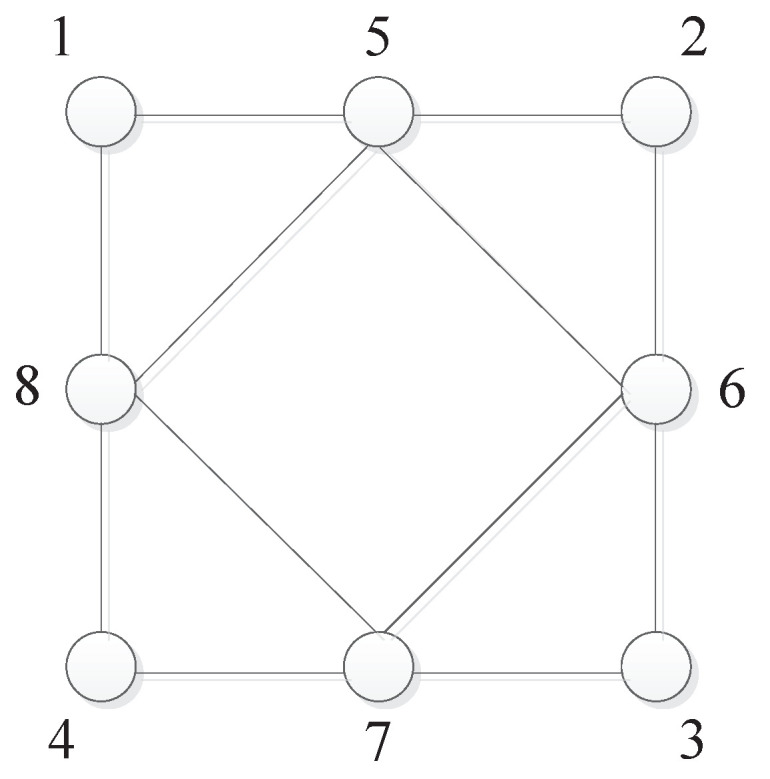
Undirected and connected topology.

**Example** **1.** *Consider a multi-agent system of eight agents (12), and the information-exchanging topology presented in Figure 1 is undirected and connected. In Figure 1, the circle denotes the agent, and the number denotes the index of the agent. For convenience, we set the adjacent weights as 1 and obtain the largest eigenvalue of L as $\lambda_{\max}=6$. The reference signals of agents are $r_{1}=3,r_{2}=6,r_{3}=4,r_{4}=5,r_{5}=7,r_{6}=2,r_{7}=3,r_{8}=4$, and we obtain the average value of reference signals as $\frac{1}{4}\sum_{i=1}^{8}r_{i}=4.25$. Additionally, the control gains are set as κ=1 and $\kappa_{I}=0.5$.*

**Figure 2:**
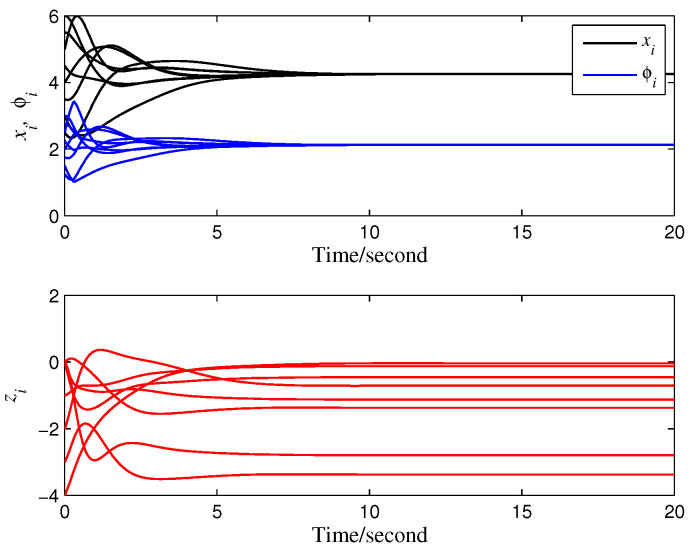
Privacy-preserving average tracking of constant reference signals.

**Figure 3:**
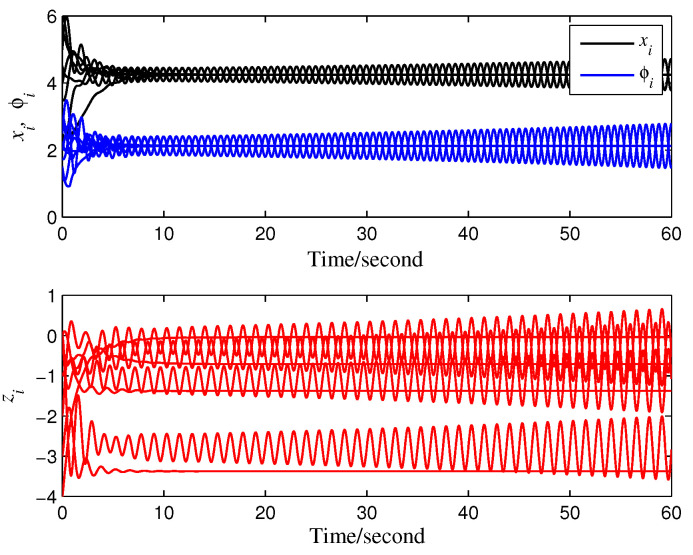
Divergence of agents’ states.

Subsequently, we set μ=0.5 and obtain $\tau_{\max}<0.723$ (s) from Theorem 1. We choose τ=0.3 (s) for convenience, and the agents reach the average value of reference signals asymptotically (see Figure 2). Obviously, the privacy of states $x_{i}\left(t\right)$ is preserved. Under a larger time delay τ=0.75 (s), the agents’ states diverge (see Figure 3), and the average value of reference signals cannot be tracked.

**Figure 4:**
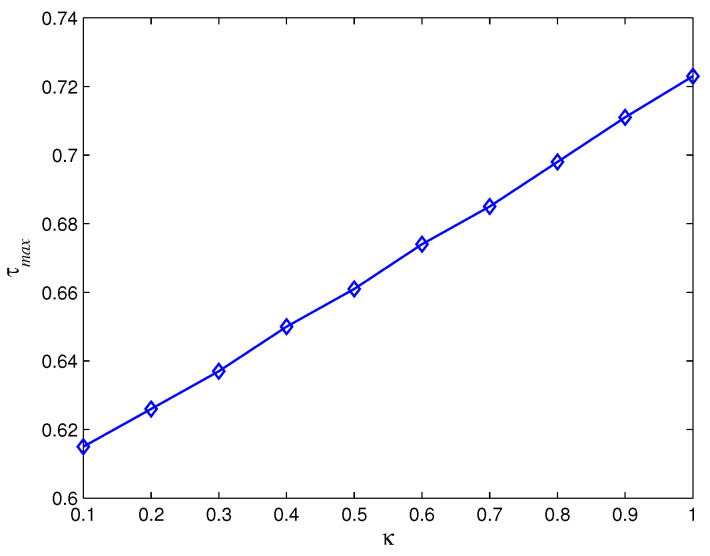
The largest time delay with different κ.

**Figure 5:**
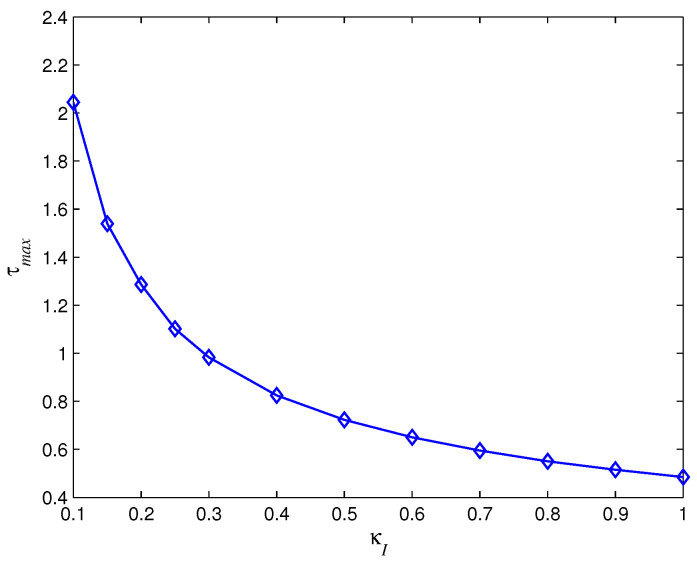
The largest time delay with different $\kappa_{I}$.

Meanwhile, we use numerical computation to analyze the sufficient and necessary conditions (3) and (14) in Theorem 1. Generally speaking, increasing the time delay prolongs the convergence time and even leads to the oscillation and divergence (see Figure 3) of agents’ states. In spite of this, different control parameters $\kappa ,\kappa_{I}$ tolerate distinct largest time delay (see Figure 4 and Figure 5). Figure 4 shows that the largest time delay $\tau_{\max}$ increases as κ increases with $\kappa_{I}=0.5,\mu =0.5$, while Figure 5 demonstrates that the largest time delay $\tau_{\max}$ decreases as $\kappa_{I}$ increases with κ=1,μ=0.5.

## 4. Average-Tracking Algorithm of Mismatched Constant Reference Signals

Motivated by the PI average-tracking algorithm in [11], a privacy-preserving average-tracking control algorithm is designed herein for first-order multi-agent systems with mismatched constant reference signals, which is formulated as follows:

(19) $$\begin{array}{ccc} u_{i}\left(t\right) & = & \delta_{i}\kappa \left(r_{i}-x_{i}\left(t\right)\right)+\kappa_{I}\sum_{j\in N_{i}}a_{ij}\left(z_{j}\left(t\right)-z_{i}\left(t\right)\right),\\ {\dot{z}}_{i}\left(t\right) & = & \sum_{j\in N_{i}}a_{ij}\left(\phi_{j}\left(t\right)-\phi_{i}\left(t\right)\right),i\in N, \end{array}$$

where κ>0, $\kappa_{I}>0$, $a_{ij}>0$ is coupling weight, $z_{i}\left(t\right)\in R$ and $\phi_{i}\left(t\right)\in R$ are auxiliary variables, and $\delta_{i}$ is defined by

$$\delta_{i}=\left\{\begin{array}{cc} 1, & \;i\in N_{1};\\ 0, & \;i\in N_{2}, \end{array}\right.$$

where $N_{1}=\left\{1,2,\cdots ,m\right\}$ with m≥2 denotes the set of valid agents that possess the reference signals, and $N_{2}=\left\{m+1,\cdots ,n\right\}$ denotes the set of extra connecting agents that have no reference signals.

With algorithm (19) and $\phi_{i}\left(t\right)=x_{i}\left(t\right)-\mu x_{i}\left(t-\tau \right)$ in (11), the closed-loop form of agents (1) is

(20) $$\begin{array}{ccc} {\dot{x}}_{i}\left(t\right) & = & \delta_{i}\kappa \left(r_{i}-x_{i}\left(t\right)\right)+\kappa_{I}\sum_{j\in N_{i}}a_{ij}\left(z_{j}\left(t\right)-z_{i}\left(t\right)\right),\\ {\dot{z}}_{i}\left(t\right) & = & \sum_{j\in N_{i}}a_{ij}\left(\left(x_{j}\left(t\right)-\mu x_{j}\left(t-\tau \right)\right)-\left(x_{i}\left(t\right)-\mu x_{i}\left(t-\tau \right)\right)\right),i\in N, \end{array}$$

where μ∈(0,1),τ>0.

The characteristic equation of system (20) for $x\left(t\right)={\left[x_{1}\left(t\right),\cdots ,x_{n}\left(t\right)\right]}^{T}$ is

(21) $$\det(s^{2}I+\kappa \Delta s+\kappa_{I}g\left(s\right)L^{2})=0,$$

where $\Delta =\mathrm{diag}\{\delta_{i},i\in N\}$.

**Theorem** **2.** *Considering the multi-agent systems (20) with an undirected information-exchanging topology satisfying Assumption 1, if*

(22) $$\tau^{2}\kappa_{I}\lambda_{\max}^{2}\frac{\left(1+\mu \right)}{\pi^{2}}<1$$

*holds, the agents (20) asymptotically track the average value of reference signals of the valid agents.*

**Proof.**  When s=0, let $\tilde{f}\left(s\right)=\det\left(s^{2}I+\kappa \Delta s+\kappa_{I}g\left(s\right)L^{2}\right)$. For the topology satisfying Assumption 1, it follows from Lemma 1 that $\tilde{f}\left(0\right)=0$ and $\tilde{f}\left(s\right)$ has only one zero at s=0. □

When s≠0, Equation (21) equals

$$\det(I+\kappa_{I}\Theta \left(s\right)L^{2})=0,$$

where $\Theta \left(s\right)=\mathrm{diag}\{{\tilde{g}}_{i}\left(s\right),i\in N\}$ with ${\tilde{g}}_{i}\left(s\right)=\frac{1-\mu e^{-s\tau }}{s(s+\kappa )},i\in N_{1}$ and ${\tilde{g}}_{i}\left(s\right)\frac{1-\mu e^{-s\tau }}{s^{2}},i\in N_{2}$.

Let $\tilde{F}\left(s\right)=\det\left(I+\kappa_{I}\Theta \left(s\right)L^{2}\right)$, and we will analyze the distribution of the zeros of $\tilde{F}\left(s\right)$. According to Lemma 2, $\tilde{F}\left(s\right)$ has its zeros on the open left-half complex plane if $\lambda (\kappa_{I}\Theta \left(j\omega \right)L^{2})$ with ω∈R does not enclose (−1,j0). Due to the symmetric weights, we obtain $L^{2}={\left(L^{2}\right)}^{T}\geq 0$. From Lemma 3, one obtains

$$\begin{array}{ccc} & & \lambda (\kappa_{I}\Theta \left(j\omega \right)L^{2})\\ & \in & \kappa_{I}\rho \left(L^{2}\right)Co\left(0\cup {\tilde{g}}_{i}\left(j\omega \right),i\in N\right)\\ & = & \kappa_{I}\lambda_{\max}^{2}Co\left(0\cup {\tilde{g}}_{i}\left(j\omega \right),i\in N\right). \end{array}$$

From Lemmas 4 and 5, all the curves ${\tilde{g}}_{i}\left(j\omega \right),i\in N$ are in the third quadrant when $\omega \in (0,\omega_{c2})$ with $\omega_{c2}=\frac{\pi }{\tau_{\max}}$, so $Co(0,{\tilde{g}}_{i}\left(j\omega \right),i\in N)$ does not enclose (−1,j0) for $\omega \in (0,\omega_{c2})$. According to the second item of Lemma 5, $|{\tilde{g}}_{i}(j\omega )|\leq \frac{1+\mu }{\omega_{c2}^{2}},i\in N$ with $\omega \in [\omega_{c2},+\infty )$. Then, the curves ${\tilde{g}}_{i}\left(j\omega \right),i\in N$ all lie in the right side of the vertical line $ℜ=-\frac{1+\mu }{\omega_{c2}^{2}}$. Hence, $Co(0,{\tilde{g}}_{i}\left(j\omega \right),i\in N)$ does not enclose $\left(-\frac{1+\mu }{\omega_{c2}^{2}},j0\right)$.

With (22), then, $\kappa_{I}\lambda_{\max}^{2}Co\left(0\cup {\tilde{g}}_{i}\left(j\omega \right),i\in N\right)$ does not enclose (−1,j0), so $\lambda (\kappa_{I}\Theta \left(j\omega \right)L^{2})$ with ω∈R does not enclose (−1,j0). Thus, the real parts of zeros of $\tilde{F}\left(s\right)$ are negative according to Lemma 2.

Hence, the roots of (21) lie in the open left-half complex plane except for one root at s=0.

Similar to the proof of Theorem 1, the states $x_{i}\left(t\right)$ and $z_{i}\left(t\right)$ of the system (4) converge to a steady state, i.e., $\lim_{t\to \infty }x_{i}\left(t\right)=x_{i}^{*},\lim_{t\to \infty }z_{i}\left(t\right)=z_{i}^{*},i\in N$. Thus, $L{\left[x_{1}^{*},\cdots ,x_{n}^{*}\right]}^{T}=0$, and it follows from Lemma 1 that $x^{*}={\left[x_{1}^{*},\cdots ,x_{n}^{*}\right]}^{T}=c1_{n}$. Thus, we obtain

$$0=\delta_{i}\kappa \left(r_{i}-x_{i}\left(t\right)\right)+\kappa_{I}\sum_{j\in N_{i}}a_{ij}\left(z_{j}\left(t\right)-z_{i}\left(t\right)\right).$$

Because of the symmetric coupling weights in Assumption 1, we obtain

$$\sum_{i=1}^{n}\delta_{i}\kappa \left(r_{i}-c\right)=0,$$

i.e.,

$$c=\frac{1}{\sum_{i=1}^{n}\delta_{i}}\sum_{i=1}^{n}\delta_{i}r_{i}=\frac{1}{m}\sum_{k=1}^{m}r_{k}.$$

Thus, the agents (20) asymptotically converge to the average value of mismatched constant reference signals. Theorem 2 is proved.

**Remark** **2.** *First-order dynamics are too simple to describe complex real systems, such as quadcopters, manipulators, etc.; therefore, high-order dynamics are adopted to express the complex plant. Referring to the design of algorithms (9) and (19), one can design the average-tracking control algorithm with matched and mismatched constant references for high-order multi-agent systems [24,25]. Additionally, the Lyapunov stability analysis method should be used to analyze the convergence property of the algorithms.*

**Figure 6:**
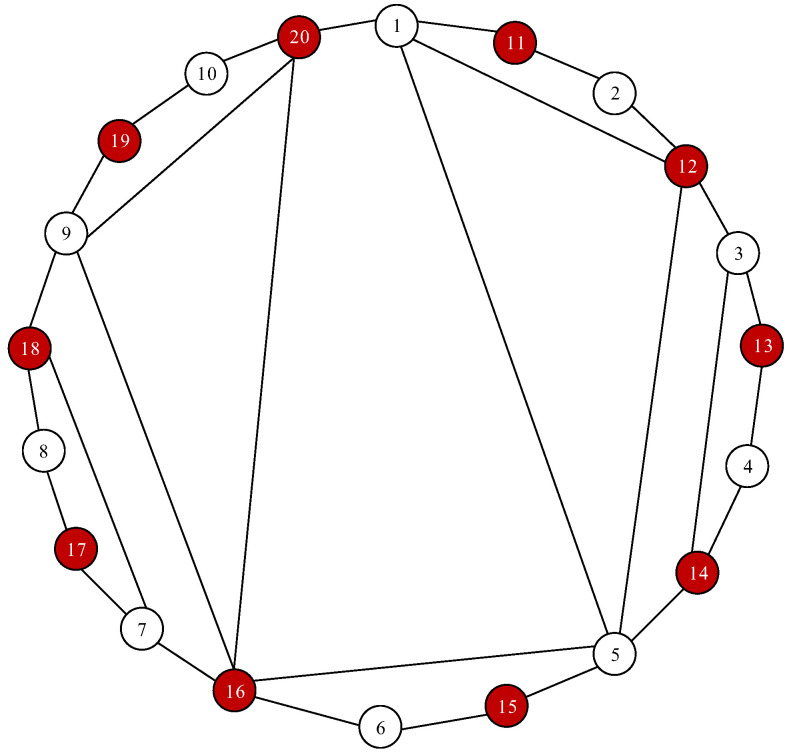
Topology of valid and extra connecting agents.

**Example** **2.** *Investigate a multi-agent system (20) of *20* agents including *10* valid agents 1,⋯,10 and *10* extra connecting agents 11,⋯,20. The information-exchanging topology adopted herein is shown in Figure 6. In Figure 6, the white circle and red circle denote the valid agents and extra connecting agents respectively, and the number denotes the index of the agent. Set the adjacent weights as *1* and obtain the largest eigenvalue of L of $\lambda_{\max}=7.1594$. The valid agents’ reference signals are $r_{1}=3,r_{2}=6,r_{3}=4,r_{4}=5$, $r_{5}=7,r_{6}=2,r_{7}=3,r_{8}=4,r_{9}=5,r_{10}=7$, so the average value of these signals is $\frac{1}{10}\sum_{i=1}^{10}r_{i}=4.6$. Additionally, the control parameters are set as κ=1, $\kappa_{I}=1$ and μ=0.5.*

**Figure 7:**
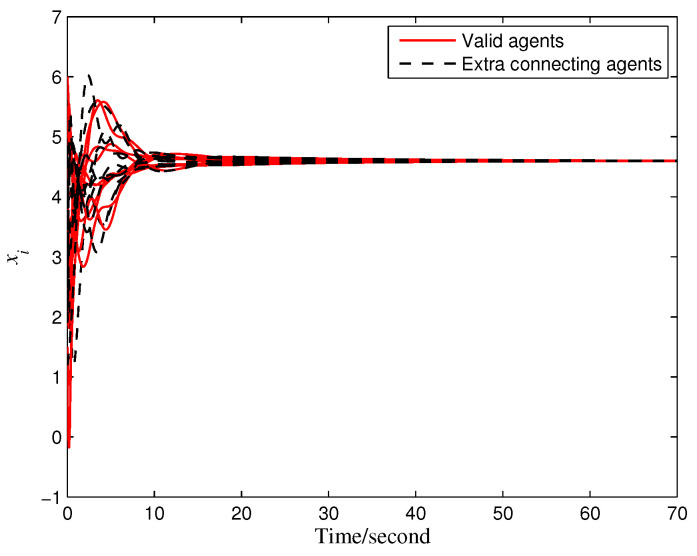
Average tracking of mismatched reference signals.

**Figure 8:**
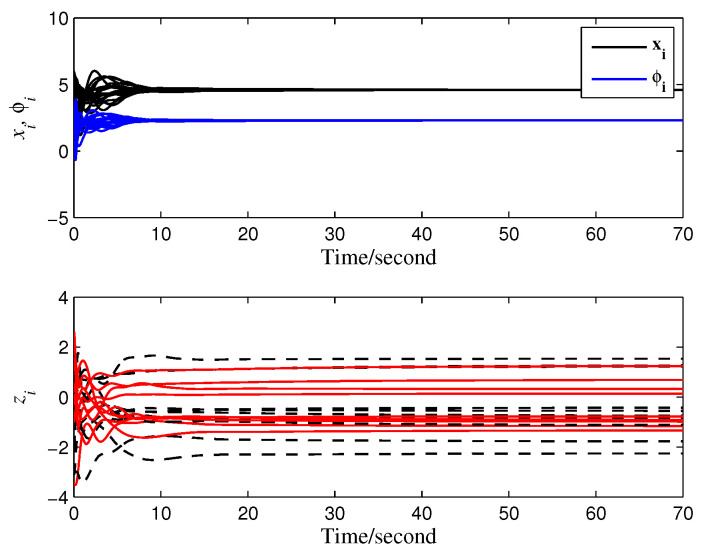
Convergence of different signals.

Accordingly, the maximum allowable time delay $\tau_{\max}=0.3583$ (s) is derived from Theorem 2. A time delay τ=0.2 (s) (satisfying (22)) is selected for simulation validation, and the results (see Figure 7) confirm that the system asymptotically converges to the average value of the mismatched reference signals. Additionally, the convergence behaviors of the distinct auxiliary and state signals illustrated in Figure 8 further verify the effectiveness of the proposed privacy preservation strategy.

## 5. Conclusions

In this study, we investigate the privacy-preserving average-tracking control problem for multi-agent systems tasked with tracking constant reference signals. By incorporating auxiliary signals that are distinct from the agents’ states and reference signals into the coordination control mechanism, an integral-type average-tracking control algorithm is proposed. Leveraging frequency-domain analysis, a delay-dependent sufficient and necessary condition is derived to guarantee that the system asymptotically tracks the average value of matched reference signals. Meanwhile, a modified privacy-preserving average-tracking algorithm is designed for multi-agent systems with mismatched reference signals, and a corresponding delay-dependent convergence condition is established via frequency-domain analysis. Our future research will extend the findings of this paper to higher-order multi-agent systems and further investigate the average-tracking control algorithm with fixed-time convergence [25] and event-triggered mechanism [26,27].

## Data Availability

The original contributions presented in this study are included in the article. Further inquiries can be directed to the corresponding author.
